# A unique case of epigastric appendicitis: a case report

**DOI:** 10.1093/jscr/rjaf246

**Published:** 2025-04-22

**Authors:** Ahmad E Al-Mulla, Mohamed Elgazzar, Omar Shalaby, Hawraa Taqi, Sulaiman Alosaimi

**Affiliations:** Department of Surgery, Farwaniya Hospital, Ministry of Health Kuwait (MOH), Sabah Al-Nasser, Block 6, PO Box 13373, Farwaniya 81004, Kuwait; Department of Surgery, Farwaniya Hospital, Ministry of Health Kuwait (MOH), Sabah Al-Nasser, Block 6, PO Box 13373, Farwaniya 81004, Kuwait; Department of Surgery, Farwaniya Hospital, Ministry of Health Kuwait (MOH), Sabah Al-Nasser, Block 6, PO Box 13373, Farwaniya 81004, Kuwait; Department of Surgery, Farwaniya Hospital, Ministry of Health Kuwait (MOH), Sabah Al-Nasser, Block 6, PO Box 13373, Farwaniya 81004, Kuwait; Department of Surgery, Farwaniya Hospital, Ministry of Health Kuwait (MOH), Sabah Al-Nasser, Block 6, PO Box 13373, Farwaniya 81004, Kuwait

**Keywords:** acute appendicitis, congenital anomalies, intestinal malrotation

## Abstract

Intestinal malrotation is a congenital condition that affects ⁓1 in every 6000 live births in which the intestines undergo abnormal rotation during early embryonic development that leads to the unusual positioning of the cecum and appendix. This abnormal placement can complicate the diagnosis of appendicitis for healthcare providers, and serious complications can result if the condition is not identified and managed promptly. This case report aims to present a rare presentation of appendicitis and to highlight the importance of understanding this congenital abnormality.

## Introduction

Acute appendicitis (AA) is a typical medical emergency. It typically presents with pain starting around the umbilicus and migrating to the right iliac fossa, commonly accompanied by symptoms such as loss of appetite, nausea, fever, and vomiting. Prompt surgical intervention is crucial to prevent complications like rupture and abscess formation. However, not all cases of appendicitis exhibit the classic presentation; some patients may experience symptoms in areas other than the right iliac fossa. Unusual presentations of appendicitis can include left lower quadrant pain [1], back pain, and groin pain due to a strangulated femoral hernia containing the appendix [2]. This case report details the unique course and management of epigastric appendicitis caused by incomplete intestinal malrotation (IM) in an adult.

## Case report

A 36-year-old male patient presented to our emergency department with a 3-day history of severe epigastric and lower abdominal pain. Four days earlier, he had experienced similar pain that was managed conservatively at his general practitioner’s clinic. The patient has no significant medical or surgical history. The pain was accompanied by repeated vomiting, nausea, anorexia, and occasional mild fever. It worsened with changes in posture and was not relieved by any painkillers or medications. Upon examination, the patient was conscious and oriented. His vital signs were as follows: temperature 37.5°C, heart rate 100 bpm, blood pressure 120/80 mmHg, and oxygen saturation 98%. Physical examination revealed generalized tenderness and guarding, particularly in the epigastric and lower abdominal regions. There was mild abdominal distension, and a digital rectal examination showed an empty rectum. No other masses or organomegaly were detected. Laboratory investigations showed a white blood cell count 14.6 (normal range: 3.9–11.1) with neutrophils at 88.3% (normal range: 39.9–72). Hemoglobin was 13.1 g/dL (normal range: 11.8–14.8). A urine test indicated the presence of nitrates, while other investigations were unremarkable.

**Figure 1:**
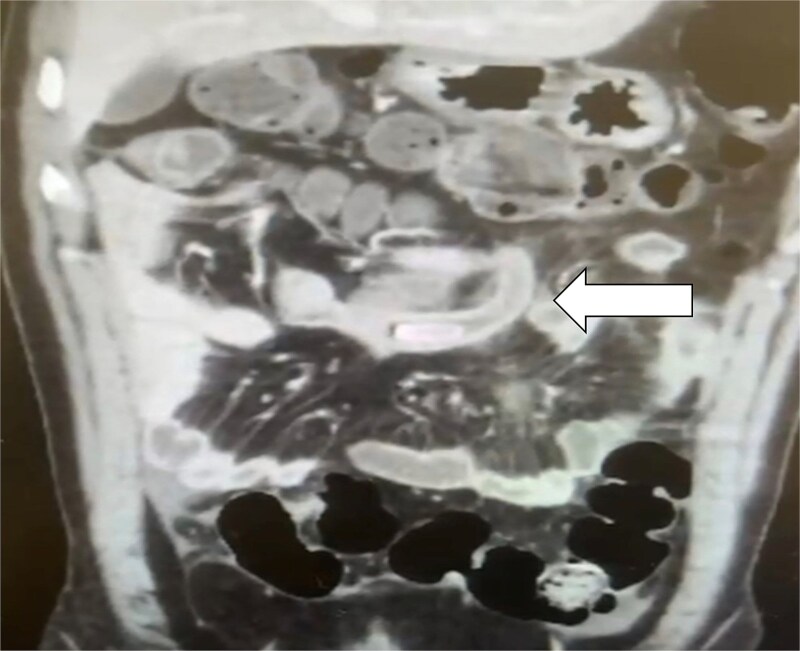
CT scan coronal view demonstrating the appendix with appendicolith at the mid-abdomen due to incomplete malrotation.

An X-ray of the abdomen showed mild distention with a few air-fluid levels; the chest X-ray was normal. The patient was admitted immediately, and a computed tomography (CT) scan was ordered. The CT scan revealed IM, indicated by the abnormal positioning of the mesenteric vessels, with the superior mesenteric vein located to the right of the superior mesenteric artery. The duodenal-jejunal junction was abnormally positioned in the right hypochondrium, while the cecum and terminal ileum were located in the mid-upper abdomen. Additionally, a dilated appendix measuring 1.2 cm, with a large appendicolith and thickened wall, was identified in the mid-upper abdomen in a sub-caecal position (see Fig. 1). The patient consented to a laparoscopic appendectomy.

### Intraoperative findings

**Figure 2:**
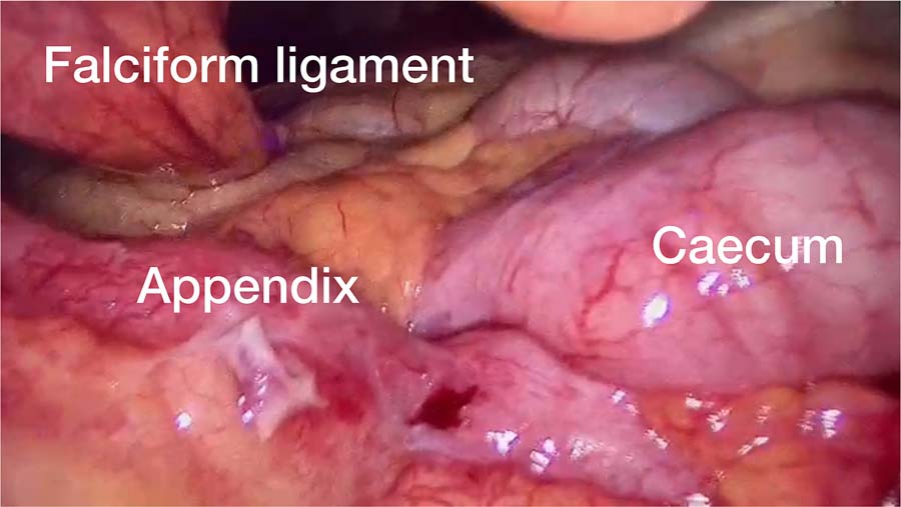
The intra-operative finding of the caecum, terminal ileum, and appendix was just below the falciform ligament.

**Figure 3:**
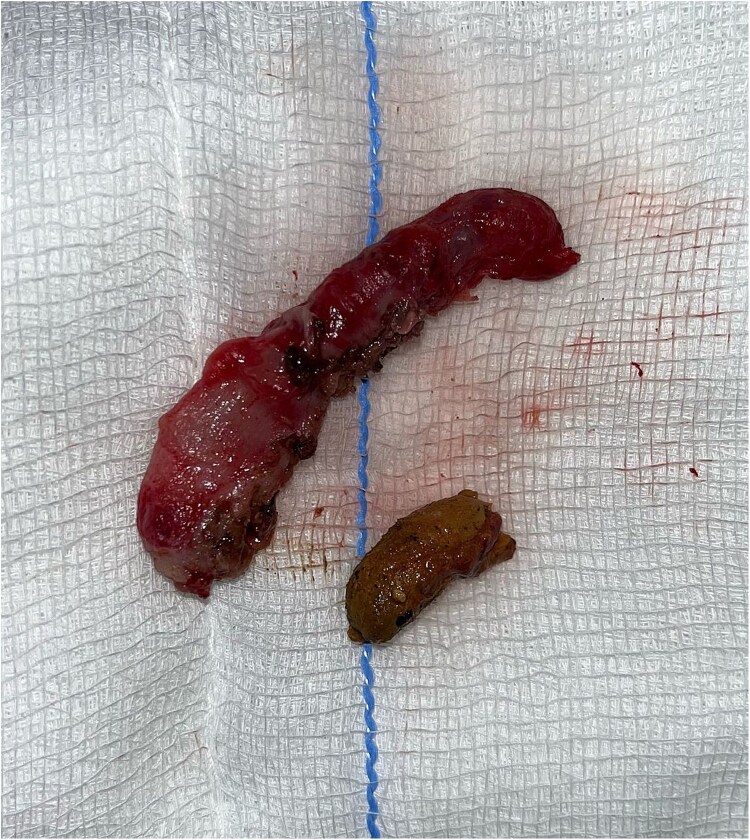
Inflamed appendix with appendicolith.

The patient was positioned supine, and an optical port was established using the Hasson technique. During the procedure, the cecum and terminal ileum were located at the center of the abdomen, just below the falciform ligament (see Fig. 2). The remaining trocars were inserted under direct visualization. The meso-appendix was dissected with a harmonic scalpel. The appendix base was ligated using an endo-loop, and the appendix and the appendicolith were excised (see Fig. 3). With a drain placed.

### Post-operative course

No adverse events were reported. The patient continued to receive antibiotics (piperacillin, tazobactam, and metronidazole) for 3 days and was discharged home after returning to a regular diet, normal bowel function, and the drain was removed. The patient reported no complaints at the 2-week follow-up, and the surgical wounds were clean.

## Discussion

AA is the most common abdominal condition, affecting ⁓1 in 400 individuals in the United States [3]. Typically, AA is diagnosed based on clinical presentation. However, in certain patients, congenital abnormalities like IM can complicate this diagnosis. About 1 in 6000 live births may present with such congenital anomalies within the first month of life, yet only 0.1%–0.5% will exhibit symptoms in adulthood, making diagnosis particularly challenging [4, 5]. The classic symptoms of AA are only observed in ⁓50% of cases, leading to a concerning misdiagnosis rate of 23.5% among adults in emergency departments [6]. This misdiagnosis is significantly more pronounced when appendicitis is associated with IM due to the abnormal position of the cecum. In our case report, the rarity of symptoms in the epigastric region made the diagnosis of appendicitis extremely challenging, highlighting the complexity introduced by such congenital anomalies.

IM is an anatomical variation resulting from incomplete intestinal rotation during weeks 4 to 12 of fetal development. While this condition can remain asymptomatic for an individual’s lifetime, it can also present as a life-threatening acute abdomen. Symptoms of IM are varied and may include intermittent colicky abdominal pain, vomiting, chronic diarrhea, and malabsorption, which tend to become more pronounced during acute episodes [7].

Several variations of IM exist, including incomplete rotation, atypical malrotation, mixed rotation, and complete rotation. IM has been alternately classified into three types: Type I (non-rotation), Type II (duodenal malrotation), and Type III (duodenal and caecal malrotation) [8].

Classical presentations or assessment tools like the Alvarado scoring system, along with focused abdominal ultrasound, considered alone can be misleading and may lead to delays in diagnosis. However, CT is a crucial tool for verifying the diagnosis in atypical cases, as demonstrated in our report. This should provide reassurance to medical professionals, as it offers a reliable method for confirming diagnoses in challenging cases and prevents delay in diagnosis, which leads to complications, as reported previously in the literature [9].

Despite the high occurrence of appendicitis associated with IM, there are currently no studies that support the practice of prophylactic appendectomy for patients who have already been diagnosed with this condition [10]. Laparoscopic appendectomy is a feasible procedure for patients with IM, offering better outcomes and shorter hospital stays, as demonstrated in our case report and supported by existing literature [8, 9]. While the open surgical approach has traditionally been used to address the malrotation, it is not necessary for adult patients.

## Conclusion

Appendicitis with malrotation is difficult to diagnose and should be considered when patient history and examination are inconsistent, especially with congenital anomalies. CT scans are essential for diagnosis. Laparoscopic surgery is safe and effective, improving visualization, recovery time, and reducing hospital stays.
